# *In vitro* and *in vivo* synergistic inhibition of *Malassezia furfur* targeting cell membranes by *Rosa rugosa Thunb.* and *Coptidis Rhizoma* extracts

**DOI:** 10.3389/fmicb.2024.1456240

**Published:** 2024-09-11

**Authors:** Li Li, Yuanyuan He, Qinghui Zou, Weiwei Chen, Yanxia Liu, Huifen He, Jun Zhang

**Affiliations:** ^1^School of Pharmaceutical Sciences, Guangzhou University of Chinese Medicine, Guangzhou, China; ^2^The First Affiliated Hospital, Guangzhou University of Chinese Medicine, Guangzhou, China

**Keywords:** *Rosa rugosa Thunb.*, *Coptidis Rhizoma*, Meilian, *Malassezia furfur*, synergistic antifungal

## Abstract

**Background:**

*Malassezia furfur (M. furfur)* is a prevalent dermatophyte that significantly impairs patients’ quality of life. This study aimed to evaluate the synergistic antifungal effects of combined extracts from *Rosa rugosa Thunb*. (MG) and *Coptidis Rhizoma* (HL) against *M. furfur*, both in vitro and in vivo.

**Methods:**

High-performance liquid chromatography (HPLC) was used to identify the major active compounds present in MG and HL. The antifungal activity of the combined Meilian extract (ML) was assessed using the checkerboard method and time-kill curves. Microstructural alterations in the fungi were observed using scanning electron microscopy (SEM) and transmission electron microscopy (TEM). The impact of the extracts on the fungal cell membrane was investigated through propidium iodide staining, protein concentration assays, and ergosterol quantification. Transcriptomic analysis was conducted to elucidate the molecular mechanisms underlying the effects of the extracts. Furthermore, the synergistic antifungal effects of ML were evaluated in a mouse model of seborrheic dermatitis induced by *M. furfur*.

**Results:**

The study demonstrated that the combined application of MG and HL significantly affected the integrity of the *M. furfur* cell membrane and potentially modulated its formation processes. In the *M. furfur*-induced seborrheic dermatitis model, ML exhibited synergistic antifungal effects and effectively alleviated skin inflammation. These findings provide an important theoretical basis for understanding the antifungal mechanisms of ML and its potential application in dermatological therapy.

## 1 Introduction

The skin microbiome is one of the largest microecosystems of the human body, essential for maintaining skin health through its intricate ecological balance (Grice and Segre, 2011; Boxberger et al., 2021). *Malassezia*, a lipid-dependent basidiomycete yeast, is a resident of human and warm-blooded animal skin, primarily growing in the stratum corneum. Its lipophilic nature significantly impacts skin ecological balance. Studies indicate that abnormal proliferation of *Malassezia* has been linked to various skin diseases, such as pityriasis versicolor (PV), Malassezia folliculitis (MF), and seborrheic dermatitis/dandruff (SD/D) (Ianiri et al., 2022; Vest and Krauland, 2023). Seborrheic dermatitis, affecting approximately 3% of the general population, is characterized by erythema, itching, and varying degrees of scaling, significantly reducing patients’ quality of life (Borda et al., 2019; Hamdino et al., 2022). *M. furfur* is considered one of the main pathogens. Various azole drugs are used to treat seborrheic dermatitis caused by *M. furfur*, but long-term use of antifungal drugs can cause irritation and resistance. Zinc pyrithione (ZPT), a commonly used treatment for seborrheic dermatitis, has been banned by the European Commission due to its potential carcinogenic, mutagenic, and teratogenic effects (Schwartz, 2016; Huang et al., 2021; Liu et al., 2022). Thus, there is an urgent need to identify new, safe, and effective natural antifungal alternatives (Ayatollahi et al., 2021).

In recent years, a variety of natural plant products, including natural compounds, plant extracts, and essential oils, have demonstrated significant antibacterial and antifungal properties. Their nontoxic nature, cost-effectiveness, and efficacy position them as promising alternatives to conventional antifungal agents (Ayatollahi et al., 2021). *Rosa rugosa Thunb.* is known for its rich content of bioactive compounds, such as flavonoids, phenolic acids, amino acids, trace elements, volatile oils, and polysaccharides (Nowak et al., 2014). Studies have shown that rose extracts can inhibit the activity of various bacteria and fungi, including *Staphylococcus epidermidis, Staphylococcus aureus, Escherichia coli, Klebsiella pneumoniae, Candida albicans,* and *Candida parapsilosis* (Nowak et al., 2014; Cendrowski et al., 2020). *Coptidis Rhizoma* is a Ranunculaceae plant containing main components such as berberine and coptisine, which possess broad-spectrum antibacterial effects (Wang et al., 2019). Research has found that coptisine exhibited strong antifungal activity, inhibiting the growth of *Candida albicans* at low concentrations (Kong et al., 2009). Furthermore, berberine hydrochloride has been reported to possess antibacterial activity against *Staphylococcus aureus*.

While MG and HL exhibit various degrees of antifungal activity, the antifungal effect of a single plant component is often limited. Consequently, the synergistic effect of different plant extracts might result in more significant therapeutic outcomes. In addition, to date, there are no reports on the synergistic antifungal effects of MG and HL against *M. furfur in vitro* and *in vivo*. This study aims to reveal the synergistic antifungal activity of MG and HL against *M. furfur*. This will be achieved by investigating the physiological and molecular mechanisms affecting cell membrane function and verifying the synergistic antifungal effects and therapeutic potential in a seborrheic dermatitis mice model induced by *M. furfur*. This study contributes to new insights into the synergistic antifungal mechanisms of ML extract and promotes its application in treating skin diseases caused by *M. furfur*.

## 2 Materials and methods

### 2.1 Materials

*Rosa rugosa Thunb.* and *Coptidis Rhizoma* were purchased from Guangzhou Zhixin Traditional Chinese Medicine Slice Co., Ltd., and identified by Professor Zhang Jun from Guangzhou University of Chinese Medicine. *Malassezia furfur* (*M. furfur*, BNCC324536) was procured from Beina biology-Henan industrial microbial strain engineering technology research center (Henan, China); ATCC Modified Dixon medium (mDixon) was acquired from Qingdao Haibo Biotechnology Co., Ltd. (Qingdao, China); Propidium Iodide (PI) fluorescent dye was sourced from Yesen Bio-tech (Shanghai, China); BCA Protein Assay Kit was obtained from Beyotime (Haimen, China). All other chemicals were purchased from Sigma-Aldrich Corporation (MO, United States).

### 2.2 Preparation of MG, HL and ML

MG and HL were prepared as follows: 25 g of each plant material were accurately weighed. A two-step extraction process was employed using 60% ethanol at a ratio of 1:6 (w/v), with each extraction lasting for 1 h. The extracts were combined, filtered, and then concentrated under reduced pressure at 60°C to a final volume of 25 mL, resulting in a crude drug concentration of 1.0 g/mL for both extracts. Prior to experimentation, the stock solutions were diluted with the growth medium to an intermediate concentration, after which a surfactant mixture was added. This mixture composed of 1 g propylene glycol, 2 g ethoxylated hydrogenated castor oil, and 0.59 g isopropyl palmitate, was added to adjust the final concentration of the solution, ensuring that the surfactant comprised 10% of the total volume of the final diluted solution.

Preparation of the ML: The diluted solutions of the MG and HL were mixed in a 1:2 ratio to yield the final ML solution.

### 2.3 Preparation of *M. furfur* suspension

*Malassezia furfur* was first streaked on ATCC mDixon agar plates and cultured at 30°C for 72 h. A single colony was selected and transferred into mDixon liquid medium, then incubated at 200 rpm, 30°C for 48 h. The cells were collected by centrifugation and washed twice with physiological saline solution. Subsequently, the turbidity of the fungal suspension was adjusted to match the McFarland standard No. 1, and the concentration, confirmed using the plate count method, was (1–2) × 10^6^ CFU/mL.

### 2.4 HPLC analysis

The analysis of the extracts was performed using an Agilent 1,260 HPLC system (Agilent Technologies, United States), equipped with a variable wavelength detector (VWD) and a Kromasil 100-5-C18 column (250 × 4.6 mm, 5 μm). The mobile phase comprised solvents A and B, utilizing a gradient elution method as follows: 2–10% B from 0 to 10 min, 10–13% B from 10 to 20 min, 13–14% B from 20 to 35 min, 14–22% B from 35 to 40 min, 22% B from 40 to 70 min, 22–50% B from 70 to 90 min. Solvent A was a 0.3% aqueous phosphoric acid solution, solvent B was acetonitrile. The detection wavelength was set at 280 nm, with a column temperature maintained at 30°C, an injection volume of 2 μL, and a flow rate of 1.0 mL/min. For sample preparation, 1 mL of each MG, HL and ML was transferred into a 10 mL volumetric flask. An adequate volume of 60% ethanol was added, followed by sonication (280 W, 40 kHz) for 30 min. The extracts were then allowed to equilibrate at room temperature and adjusted to the final volume with ethanol. Afterward, the samples were filtered. The HPLC analysis was conducted as described, and the compounds gallic acid, rutin, coptisine, palmatine, epiberberine and berberine were analyzed using the aforementioned methodology.

### 2.5 *In vitro* antifungal activity against *M. furfur*

#### 2.5.1 Determination of minimum inhibitory concentration

To ascertain the antifungal activity of MG and HL against *M. furfur*, the broth microdilution method was employed to determine the minimum inhibitory concentration (MIC) (Wang S. et al., 2024). Initially, the fungal strains were cultured to the logarithmic growth phase, followed by the preparation of serial dilutions of extracts in a 96 well plate. Each well received 100 μL of fungal suspension mixed with 100 μL of the drug solution, including both positive and negative controls. The 96well plates were incubated at 30°C for 72 h. The lowest inhibitory concentration at which no visible colonies appeared to the naked eye was defined as the MIC.

#### 2.5.2 Determination of fractional inhibitory concentration index (FICI)

Microdilution checkerboard test was used for determining the FICI of antifungal combination of MG and HL, with some modifications (White et al., 1996). The antifungal agents were tested within a concentration range of 1/64 MIC to 4 MIC, while the concentration of *M. furfur* suspension remained the same as previously described. Fractional inhibitory concentration (FIC) and fractional inhibitory concentration index (FICI) were calculated as follows: FIC = MIC of a drug in combination/MIC alone; FICI = FICA + FICB. FICI values of≤0.5 indicates synergistic activity, 0.5 < FICI≤1 indicates additive activity, 1 < FICI≤2 indicates indifferent interactions, and FICI≥2 indicates antagonistic interactions. All experiments were performed in triplicate.

#### 2.5.3 Time-kill curve determination

The time-kill curve of MG and ML against *M. furfur* was determined using the method with minor modifications (Chen Z. et al., 2022). *M. furfur* in the logarithmic growth phase was exposed to treatments with MG at a crude drug concentration of 3.125 mg/mL, HL at 6.25 mg/mL, and ML at the MIC concentration, and then incubated at 30°C with shaking at 200 rpm. At predetermined time points (0 h, 3 h, 6 h, 12 h, 24 h, 36 h, 48 h, 72 h), aliquots of 100 μL from the fungal suspension were taken, serially diluted with sterile saline, and plated on solid medium. After incubation at 30°C for 72 h, the colony count was recorded. The time-kill curves were plotted with time as the x-axis and inhibition rate as the y-axis.

### 2.6 Ultrastructure observation of *M. furfur*

#### 2.6.1 Scanning electron microscopy (SEM)

SEM was used to observe the morphological changes in *M. furfur* following treatment with MG and HL (Chen J. et al., 2022). *M. furfur* suspension (10^6^ CFU/mL) was inoculated in medium containing MG at a crude drug concentration of 3.125 mg/mL, HL at 6.25 mg/mL, and ML at the MIC concentration, with untreated controls. After 48 h of incubation at 37°C, the fungi were collected by centrifugation, washed with PBS, and fixed with electron microscopy fixative at 4°C for 12 h. After washing with PBS, the samples were dehydrated through a series of ethanol solutions (30, 50, 70, 80, 90, 95, and 100%), treated with a mixture of ethanol and isoamyl acetate (v/v = 1/1), and then with pure isoamyl acetate. The samples were then critically point dried, mounted on sample stubs using conductive adhesive, gold-coated, and observed under a scanning electron microscope.

#### 2.6.2 Transmission electron microscopy (TEM)

TEM was employed to observe the ultrastructural changes in *M. furfur* treated with MG at a crude drug concentration of 3.125 mg/mL and HL at 6.25 mg/mL, and ML at the MIC concentration (Zhou et al., 2024). The sample preparation was the same as for SEM. After dehydration with ethanol, the samples were immersed in pure acetone for 20 min followed by infiltration with a graded series of embedding agent and acetone mixtures (1,3, 1:1, and pure embedding agent) at room temperature. The samples were embedded in embedding molds and polymerized at 37°C for 24 h, followed by 48 h at 60°C. Ultrathin sections (100 nm) were obtained using an ultramicrotome, stained with uranyl acetate in 50% ethanol and lead citrate, and finally observed under a transmission electron microscope.

### 2.7 Effect on *M. furfur* cell membrane

#### 2.7.1 Propidium iodide fluorescence staining

To evaluate changes in cell membrane integrity of *M. furfur* following treatment with MG at a crude drug concentration of 3.125 mg/mL and HL at 6.25 mg/mL, and ML at the MIC concentration, Propidium Iodide (PI) fluorescence staining was employed (Wang et al., 2023). PI, a cell impermeant dye, is utilized to evaluate cell permeability. *M. furfur* suspension (100 μL) was incubated with MG, HL, and ML in 6well plates at 30°C for 24 h. Following incubation, the cells were centrifuged and washed with PBS. The cells were resuspended in 500 μL of Binding Buffer, and 3 μM PI was added and incubated in the dark for 10 min. The samples were washed with PBS, resuspended in PBS buffer, and observed under a fluorescence microscope. The excitation and emission wavelengths of PI were set to 535 nm and 617 nm, respectively.

#### 2.7.2 Protein concentration measurement

The total protein content in *M. furfur* cells treated with MG at a crude drug concentration of 3.125 mg/mL and HL at 6.25 mg/mL, and ML at the MIC concentration was determined (Wang L. et al., 2024). *M. furfur* suspension (10^6^ CFU/mL) was incubated in 6well plates for 24 h, followed by centrifugation, and washing with PBS. Subsequently, the cell density was adjusted, and the appropriate concentrations of MG, HL, and ML were added. After 48 h of incubation at 30°C, the cells were collected, washed with PBS, and lysed with RIPA lysis buffer (containing protease inhibitors) with a homogenizer. The lysate was centrifuged, and the supernatant was used for protein content measurement using the BCA protein assay kit.

#### 2.7.3 Ergosterol content measurement

The effect of MG and HL on ergosterol content in *M. furfur* was measured using high performance liquid chromatography (HPLC) (OuYang et al., 2021). *M. furfur* suspension (100 μL) was incubated with MG at a crude drug concentration of 3.125 mg/mL and HL at 6.25 mg/mL, and ML at the MIC concentration in 6well plates at 30°C for 72 h, centrifuged, and washed. The fungal cells were weighed, and 15 mL of ethanol KOH solution was added. The mixture was vortexed, sonicated, and incubated at 80°C for 2 h. After cooling to room temperature, saponified products were extracted with petroleum ether and washed with distilled water. The petroleum ether layer was evaporated, and the unsaponifiable fraction was dissolved in methanol, filtered through a 0.22 μm filter, and analyzed by HPLC.

### 2.8 Transcriptomics

#### 2.8.1 Sample preparation

*Malassezia furfur* in the logarithmic growth phase was diluted to 10^6^ CFU/mL and incubated with MG at a crude drug concentration of 3.125 mg/mL and HL at 6.25 mg/mL, and ML at the MIC concentration in sterile, enzyme free 6well plates at 30°C for 72 h. The cells were collected by centrifugation, washed with PBS, and immediately frozen in liquid nitrogen and stored at 80°C. Total RNA was extracted using Trizol reagent and the purity, quality, and integrity were assessed with a spectrophotometer.

#### 2.8.2 cDNA library construction and sequencing

Total RNA was isolated using Trizol Reagent (Invitrogen Life Technologies) and quantified using a NanoDrop spectrophotometer (Thermo Scientific). cDNA libraries were constructed from 3 μg of RNA as input material, following standard protocols. mRNA was purified using poly-T oligo magnetic beads, fragmented, and reverse transcribed using random hexamers and Superscript II. The second strand cDNA synthesis was performed using DNA Polymerase I and RNase H, followed by end repair, A-tailing, adapter ligation, and PCR amplification. The libraries were purified using AM-Pure XP beads and assessed using the Agilent 2,100 bioanalyzer. Sequencing was performed on an Illumina platform.

#### 2.8.3 Bioinformatics analysis of transcriptome sequencing data

High quality reads were obtained by filtering raw data using fastp (v0.22.0). Clean reads were aligned to the *M. furfur* reference genome using HISAT2 (v2.1.0), and gene expression was quantified using HTSeq (v0.9.1). Differential expression analysis was conducted using DESeq (v1.38.3), with the following criteria: |log2FoldChange| > 1 and *p* < 0.05. GO and KEGG enrichment analyses were performed to annotate and analyze the functions of differentially expressed genes (DEGs).

### 2.9 Effect of ML extract on *M. furfur*-induced seborrheic dermatitis in mice

A seborrheic dermatitis mice model was established using *M. furfur* olive oil suspensions (Yang et al., 2022). SPF Balb/c male mice were randomly divided into seven groups: control, model, MG (0.1 g/mL), HL (0.2 g/mL), low-dose ML (a combination of 0.1 g/mL MG + 0.2 g/mL HL), high-dose ML (a combination of 0.4 g/mL MG + 0.8 g/mL HL), and ketoconazole (0.08 mg/mL), with eight mice per group. Before the experiment, the back hair of the mice was removed using depilatory cream, and the skin barrier was disrupted with sandpaper for two consecutive days. 200 μL of *M. furfur* olive oil suspension (10^9^ CFU/mL) was applied to the back skin of the mice for eight consecutive days, while the control group received olive oil. Each group of mice was treated with the corresponding extract for the study duration. Skin scales were collected on days 0, 7, and 14, cultured on solid plates, and the fungal colony count was recorded. On day 14, the mice were euthanized, and skin samples were collected for hematoxylin and eosin (HE) staining. This study was approved by the Animal Ethics Committee of Guangzhou University of Chinese Medicine.

### 2.10 Statistical analysis

SPSS 26.0 software was used for statistical analysis. The Shapiro Wilk test was employed to check the normality of the data. If the data followed a normal distribution, the results were expressed as mean ± standard deviation. Group comparisons were performed using one-way ANOVA with Levene’s test for homogeneity of variances. When variances were homogeneous, LSD tests were used for multiple comparisons. When variances were heterogeneous, Dunnett’s T3 test was applied. A *p*-value <0.05 was considered statistically significant.

## 3 Results

### 3.1 HPLC analysis

**Figure 1:**
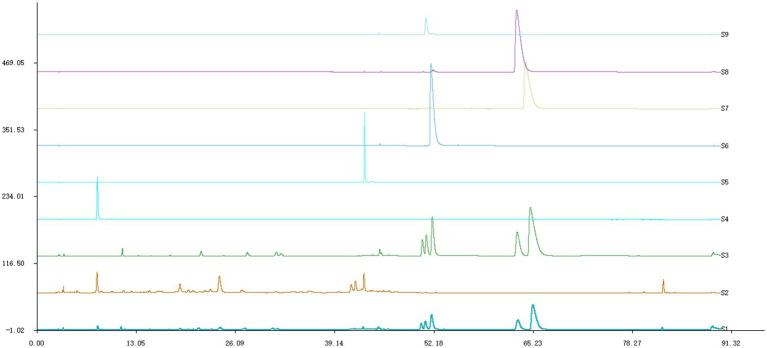
HPLC chromatogram. S1 was ML, S2 was MG, S3 was HL, S4 was gallic acid, S5 was rutin, S6 was coptisine, S7 was berberine, S8 was palmatine,S9 was epiberberine.

After identification, ML was found to contain six components (Figure 1). Gallic acid, rutin belong to MG, while berberine, palmatine, epiberberine and coptisine belong to HL.

### 3.2 Antifungal activity of MG and HL against *M. furfur*

#### 3.2.1 MIC and FICI determination

The results indicated that the MICs of MG and HL against *M. furfur* were 12.5 mg/mL and 25 mg/mL, respectively. Interestingly, when combined, referred to as the Meilian (ML), a combination of crude drug concentration of 3.125 mg/mL of MG and 6.25 mg/mL of HL achieved the MIC, with an FICI value of 0.5, indicating a synergistic effect. Specific results are presented in Tables 1, 2.

**Table 1:** Antifungal activity of MG and HL against *M. furfur*.

Drugs	MIC (mg/mL)
MG	12.5
HL	25
Ketoconazole	3.125 × 10^-3^
Surfactant	>250

**Table 2:** FICI determination of MG and HL against *M. furfur*.

Drugs	Alone MIC (mg/mL)	Combined MIC (mg/mL)	FICI	Interaction relationship
MG	12.5	3.125	0.5	Synergistic
HL	25	6.250		

#### 3.2.2 Time-kill curve

**Figure 2:**
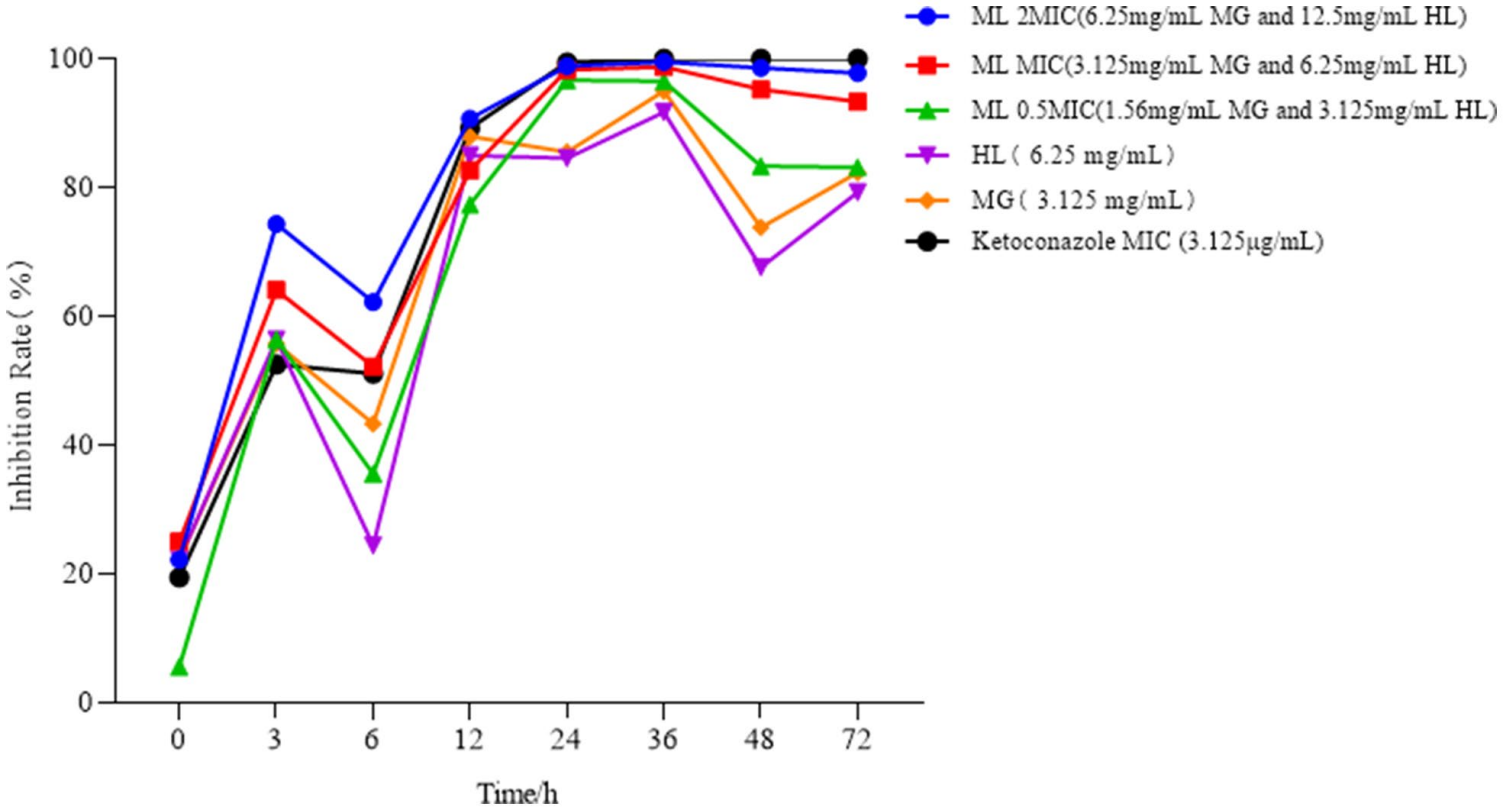
Effect of treatment with MG, HL, and ML on the time-kill curve of *M. furfur*.

As illustrated in Figure 2, the growth of *M. furfur* was inhibited to varying extents following treatment with MG and HL, either individually or in combination. At the 3-h mark, the inhibition rate in all treatment groups exceeded 50%. At 6 h post-treatment, the growth rate of the control group surpassed the rate at which the drug inhibited the fungus, resulting in a decreased inhibition rate. By 12 h, the inhibition rate in all treatment groups was above 77%, and after 24 h, the rate increased to over 84%, with the combined MG and HL group reaching an inhibition rate of over 96%. At 48 h post-treatment, the control group entered a rapid growth phase, while the antifungal effects of the MG and HL when used alone were limited, leading to a reduction in the inhibition rate. Notably, the combined treatment of MG and HL at the MIC showed superior inhibitory effects compared to the individual use of each extract. Moreover, the inhibitory effect of the combined extracts on *Malassezia furfur* exhibited dose-dependence, with the effect increasing as the concentration increased. This suggests a synergistic action between the extracts of MG and HL in inhibiting the growth of *M. furfur*, with the combined use demonstrating a more pronounced antifungal effect than when used individually. These findings are consistent with the results of the FICI experiments.

### 3.3 Ultrastructure observation of *M. furfur*

#### 3.3.1 SEM

**Figure 3:**
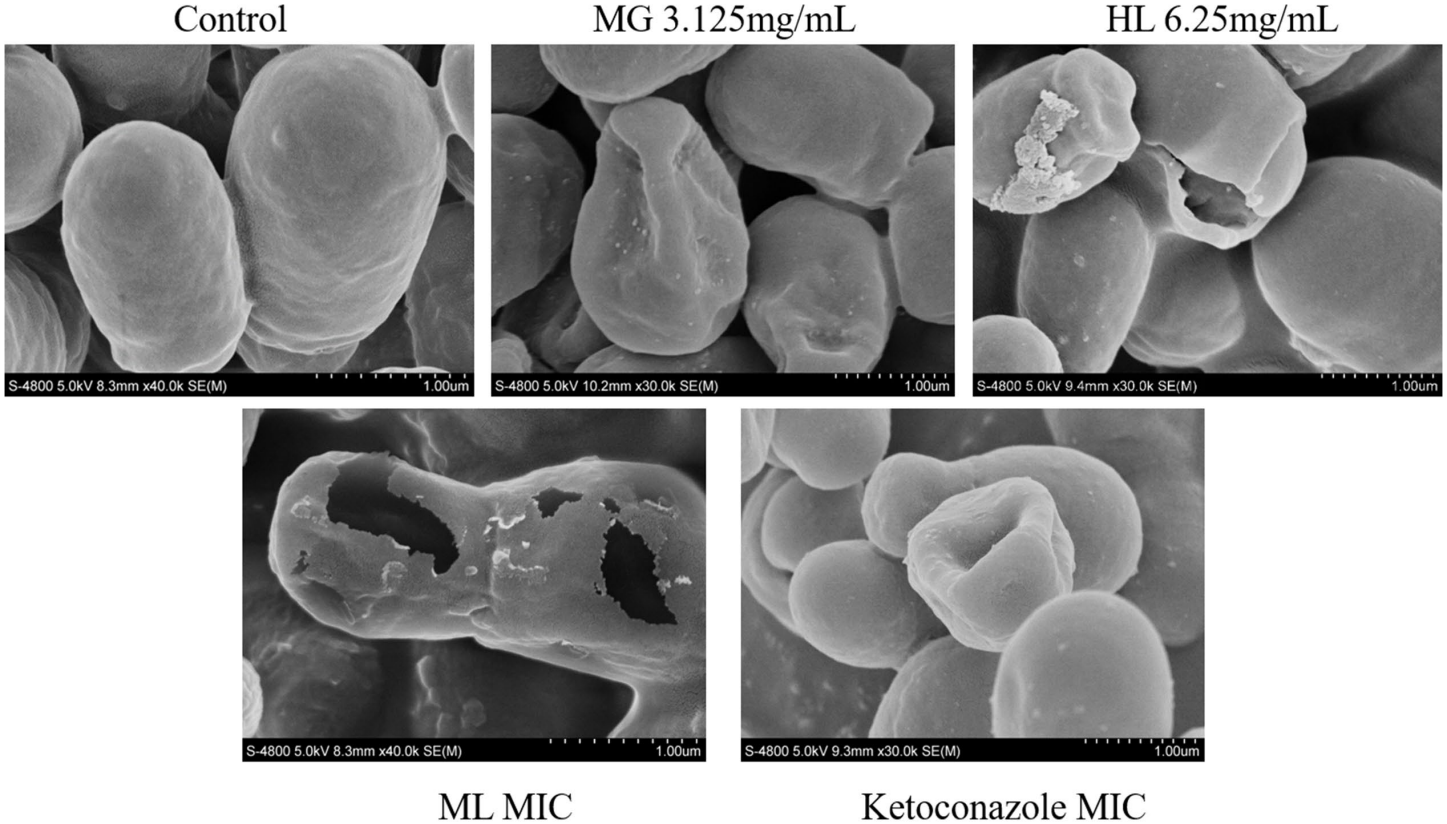
SEM images of *M. furfur* treated with MG, HL, and ML.

Figure 3 presents SEM images of *M. furfur* cells treated with MG, HL, and ML. The control group exhibited intact, oval-shaped cells with clear cell surfaces and full, dense protoplasts, with some budding states observed. In contrast, after treatment, the cells displayed irregular shapes, shrinkage, depressions, as well as varying degrees of surface holes and damage, with leakage of cellular contents. Notably, the ML group showed more severe holes and damage than the individual MG and HL groups, suggesting a potential synergistic effect in disrupting the cell wall membrane integrity of *M. furfur*.

#### 3.3.2 TEM

**Figure 4:**
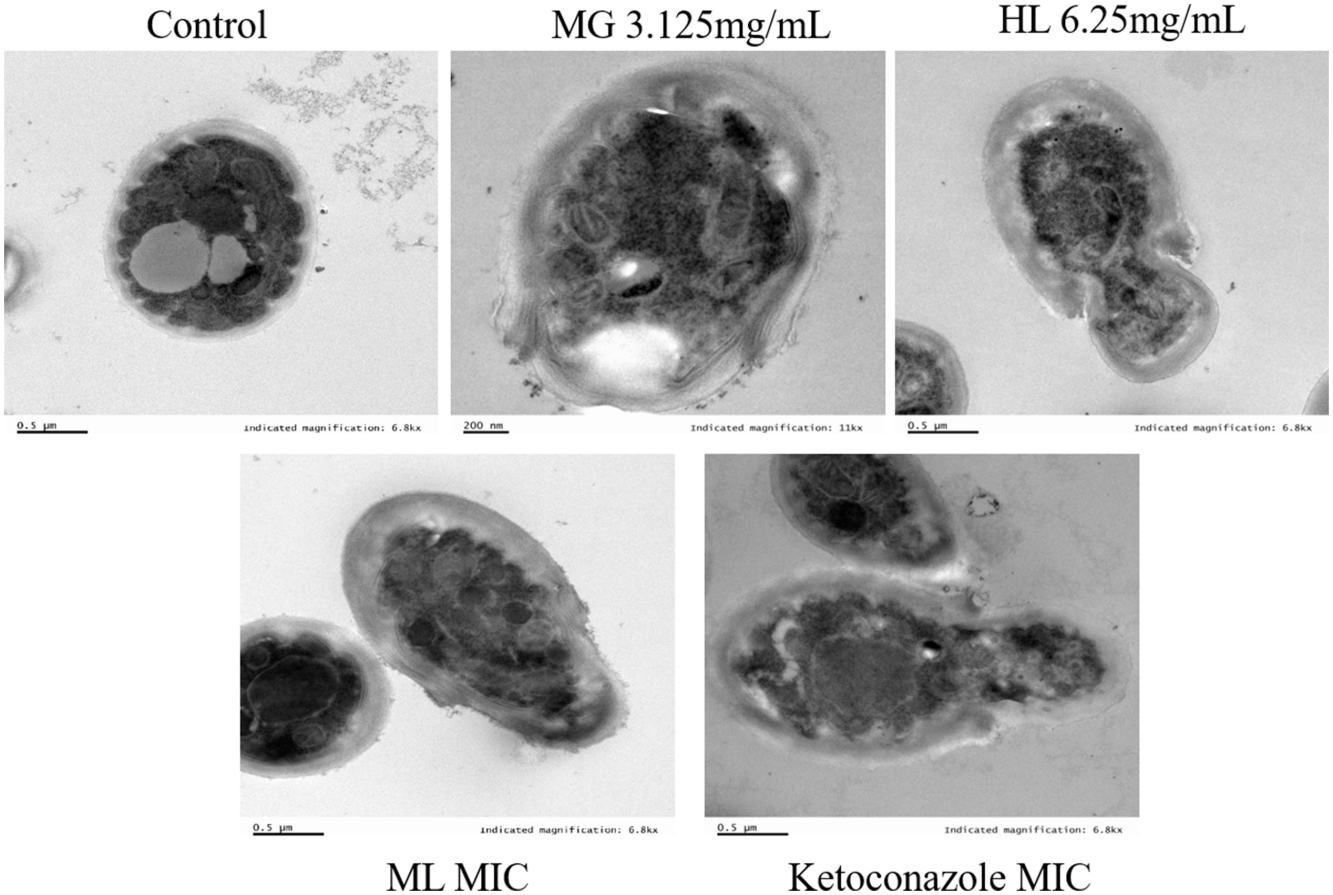
TEM images of *M. furfur* treated with MG, HL, and ML.

Figure 4 displays TEM images of *M. furfur* cells treated with MG, HL, and ML. The control group exhibited intact oval shaped cells with normal cell wall thickness, regular and complete cytoplasmic membranes, orderly cellular morphology, and intact organelles. However, after treatment, significant ultrastructural changes were observed, including increased cell volume, disrupted cell wall integrity, ruptured or thickened cytoplasmic membranes, reduced lipid droplets, disorganized nuclear structures, and swollen mitochondria with disordered internal cristae. These results suggest that MG and HL synergistically inhibit *M. furfur* growth by compromising the integrity of both the cell wall and membrane, consistent with SEM observations.

### 3.4 Effect on *M. furfur* cell membrane

#### 3.4.1 PI staining

**Figure 5:**
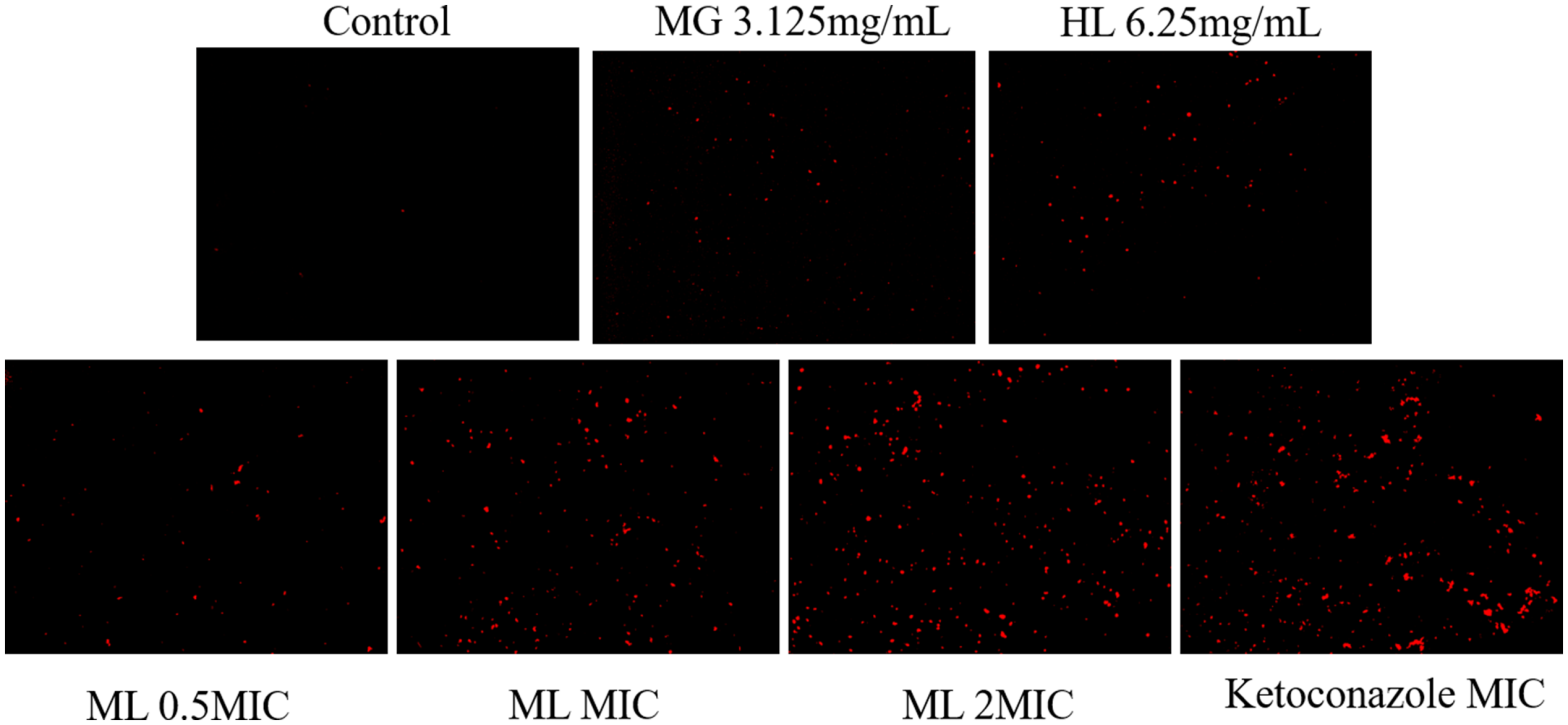
PI staining images of Effect of *M. furfur* treated with MG, HL, and ML (x10).

Disruption of cell membrane structure is a common antifungal mechanism. PI, a membrane impermeable dye, is capable of penetrating damaged cell membranes and binding to DNA, resulting in an increase in fluorescence intensity. This property allows the assessment of membrane damage by observing fluorescence intensity changes under a fluorescence microscope. As shown in Figure 5, the control group showed weak red fluorescence, signifying that PI did not penetrate the intact cell membrane, and most cells remained viable. After treatment, some cells exhibited red fluorescence, indicating varying degrees of membrane damage. The red fluorescence intensity increased with higher ML concentrations, suggesting that these extracts inhibit the growth of *M. furfur* by disrupting the integrity of the cell membrane.

#### 3.4.2 Intracellular protein content

**Figure 6:**
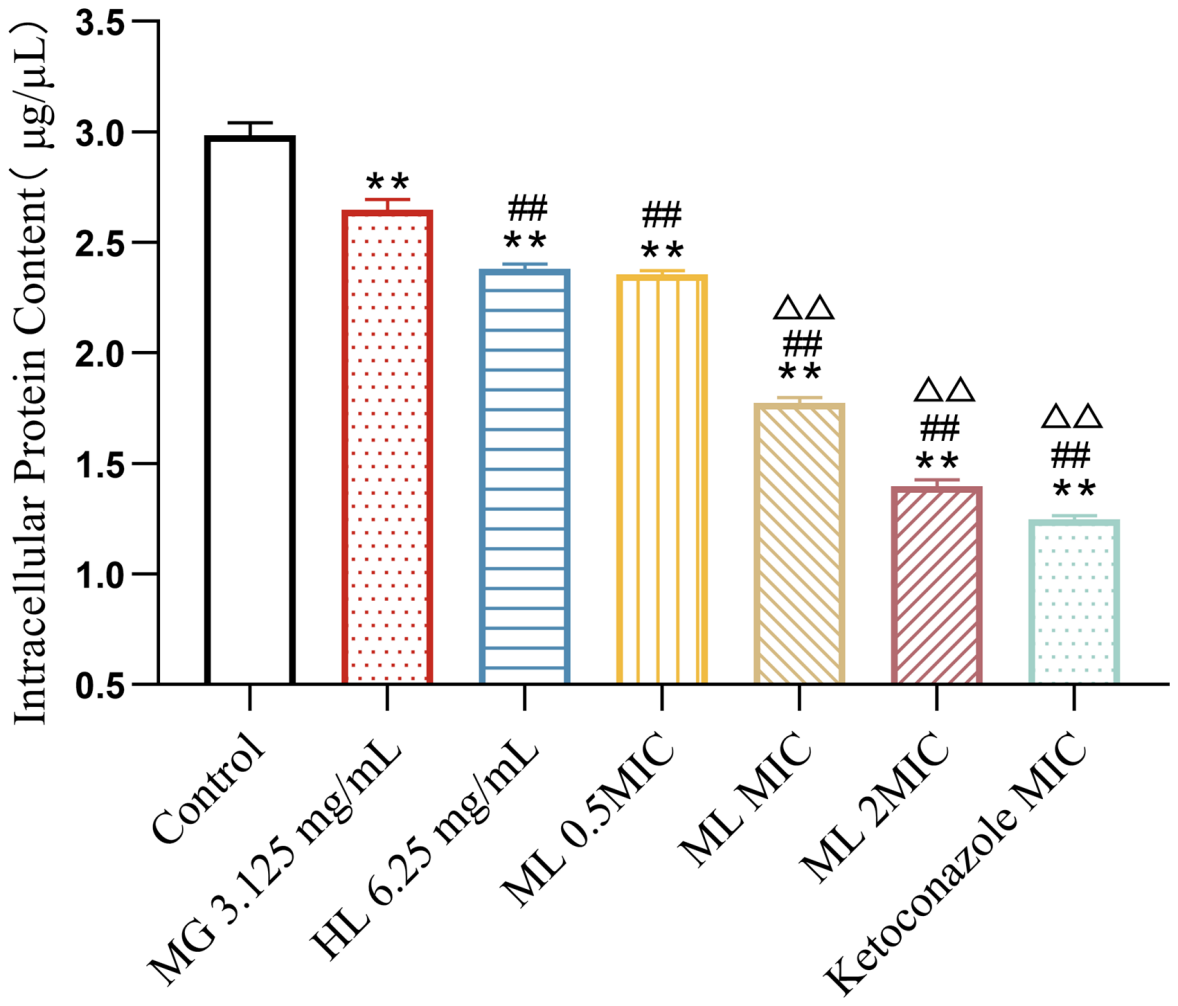
Effect of intracellular protein content of *M. furfur* treated with MG, HL, and ML (compared to control, ** *p* < 0.01; compared to the MG 3.125 mg/mL group, ^#^*p* < 0.05, ^##^*p* < 0.01; compared to HL 6.25 mg/mL group, ^△△^*p* < 0.01).

BCA protein assay results (Figure 6) revealed that a significant decrease in intracellular protein levels among groups treated with either individual or combined MG and HL extracts, compared to the control group (*p* < 0.05). Notably, treatment with MIC and 2 × MIC ML led to more pronounced protein leakage compared to individual treatments (*p* < 0.05). Specifically, the MIC ML group displayed a 29.27% increase in ergosterol inhibition rate compared to the MG group, and a 20.31% increase compared to the HL group. These findings indicate that combined treatment is more effective in disrupting cell membrane integrity, ultimately leading to protein leakage.

#### 3.4.3 Ergosterol content

**Figure 7:**
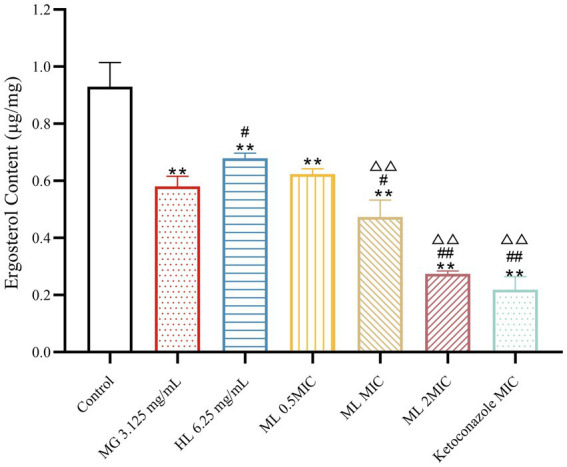
Effect of ergosterol content of *M. furfur* treated with MG, HL, and ML (compared to control, ** *p* < 0.01; compared to the MG 3.125 mg/mL group, ^#^*p* < 0.05, ^##^*p* < 0.01; compared to HL 6.25 mg/mL group, ^△△^*p* < 0.01).

Experimental results (Figure 7) demonstrated that treatment with individual and combined MG and HL significantly inhibited ergosterol synthesis in *M. furfur*. Combined treatment with MIC and 2 × MIC ML led to more significant inhibition of ergosterol synthesis (*p* < 0.05). Compared to the MG group, the MIC ML group showed a 19.69% increase in ergosterol inhibition rate, and compared to the HL group, the MIC ML group showed a 30.34% increase in ergosterol inhibition rate, suggesting that combined treatment more effectively inhibits ergosterol synthesis.

### 3.5 Transcriptomics

#### 3.5.1 Differential gene expression

**Figure 8:**
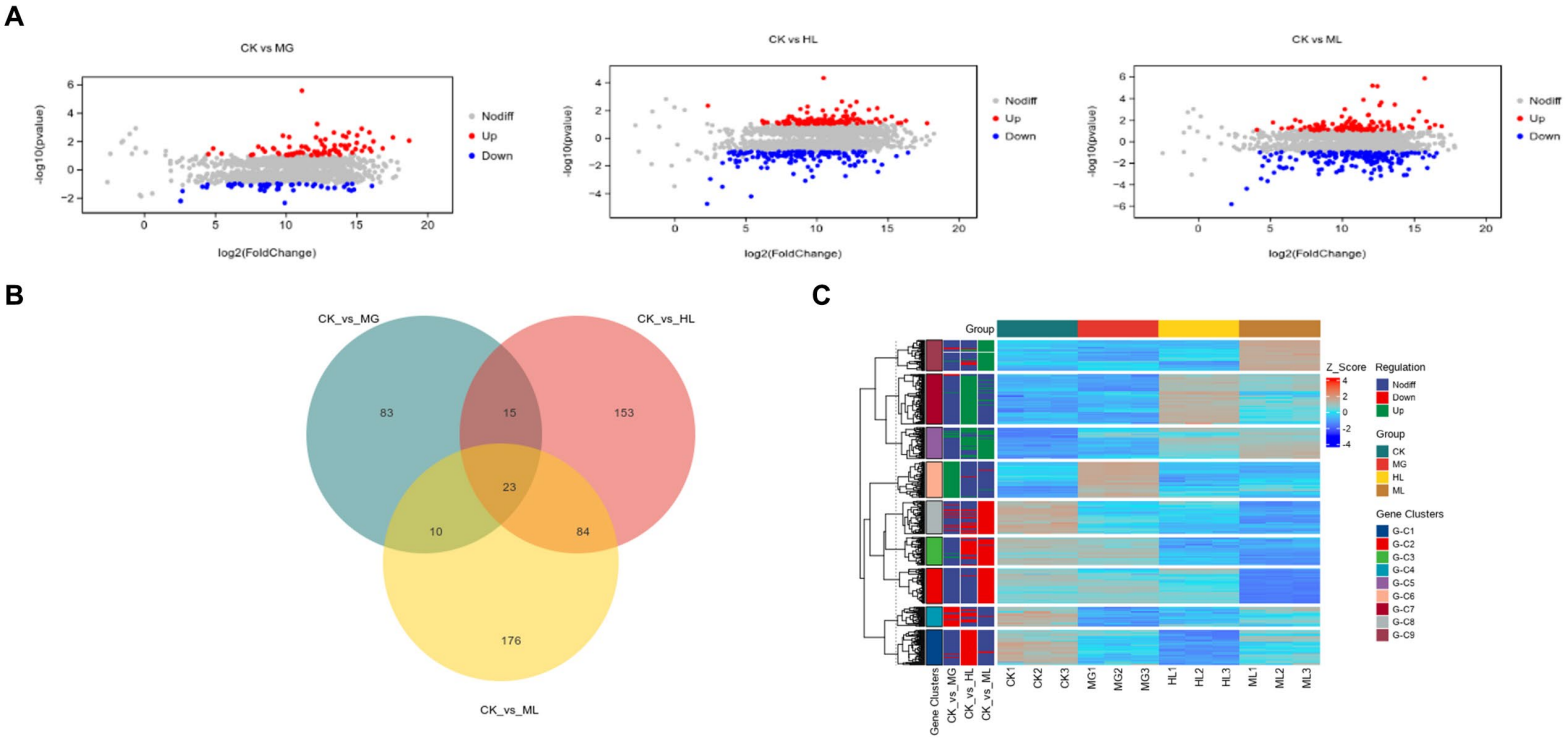
Transcriptomic analysis. **(A)** MA Plot of differentially expressed genes. **(B)** Venn diagram demonstrating the overlap of DEGs. **(C)** Heatmap of DEGs.

Compared to the control group (CK), the MG group had 131 differentially expressed genes (80 upregulated, 51 downregulated), the HL group had 275 differentially expressed genes (126 upregulated, 149 downregulated), and the ML group had 293 differentially expressed genes (116 upregulated, 177 downregulated) (Figure 8A). Venn diagrams showed 33 common differentially expressed genes between the MG and ML groups, 107 common differentially expressed genes between the HL and ML groups, and 23 common differentially expressed genes among the MG, HL, and ML groups (Figure 8B). Heatmaps indicated distinct gene expression profiles for each treatment compared to the control group, with similar expression profiles for the HL and ML groups (Figure 8C).

#### 3.5.2 GO and KEGG enrichment

**Figure 9:**
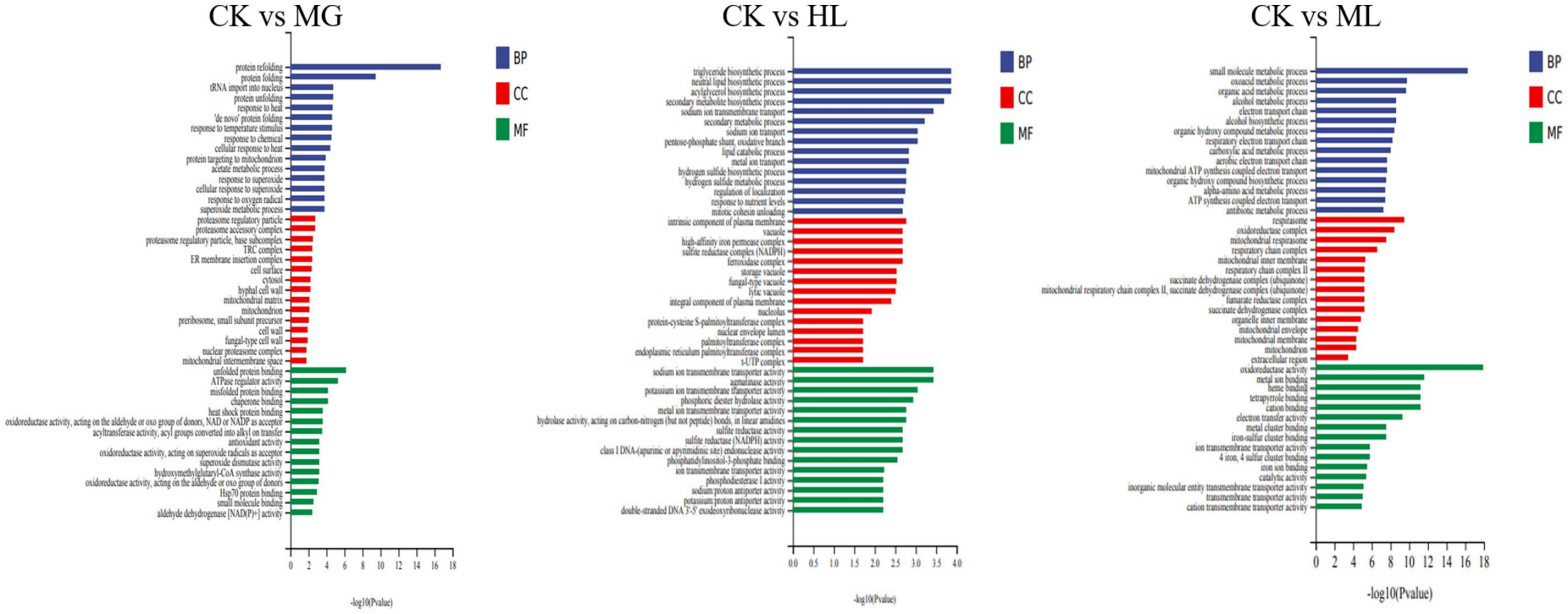
GO enrichment analysis of differentially expressed genes in *M. furfur* treated with MG, HL, and ML.

GO enrichment analysis categorized gene functions into molecular function (MF), cellular component (CC), and biological process (BP). Results indicated that most differentially expressed genes were related to ATP generation, redox reactions, and metal ion binding in MF; membrane function and mitochondrial function in CC; and small molecule metabolism and secondary metabolite biosynthesis in BP (Figure 9).

Enrichment of differential genes associated with membrane function revealed that four genes related to membrane structure were downregulated in the MG group; thirteen genes were downregulated in the HL group; eleven genes were downregulated in the ML group. The specific results are presented in Table 3.

**Table 3:** Differentially expressed genes associated with membrane function.

Group	Gene ID	Gene name	Log2 fold change	*p* values	Significant
MG	GLX27_001793	SID2	0.4731	1.08	Down
	GLX27_004576	Fgd2	0.4962	1.011	Down
	GLX27_002096	GIT4	0.3606	1.471	Down
	GLX27_003060	Bfr1	0.4822	1.052	Down
HL	GLX27_004282	Fer2	0.1167	3.1	Down
	GLX27_004283	Fer1	0.2338	2.097	Down
	GLX27_002096	GIT4	0.4367	1.195	Down
	GLX27_004596	PrnB	0.3077	1.7	Down
	GLX27_004306	Crf2	2.5137	1.3298	Up
	GLX27_000992	Ctr4	2.3102	1.208	Up
	GLX27_001106	Sir1	0.4953	1.014	Down
	GLX27_004422	MET10	0.4552	1.135	Down
	GLX27_002070	Scap	0.4999	1	Down
	GLX27_004216	PPX1	0.3358	1.574	Down
	GLX27_000894	Xpr1	0.4894	1.031	Down
	GLX27_001705	Gap1	0.4724	1.082	Down
	GLX27_001154	ERG1	0.3588	1.479	Down
ML	GLX27_003033	Sdh3	0.2608	1.939	Down
	GLX27_000123	SDH4	0.2824	1.824	Down
	GLX27_002043	QCR9	0.3759	1.412	Down
	GLX27_003437	SDH2	0.2905	1.783	Down
	GLX27_001715	Caf5	0.4249	1.235	Down
	GLX27_004596	PrnB	0.09002	3.474	Down
	GLX27_003164	Erd1	0.4645	1.106	Down
	GLX27_001154	ERG1	0.3647	1.455	Down
	GLX27_001352	ERG5	0.4547	1.137	Down
	GLX27_003903	ERG6	0.2428	2.042	Down
	GLX27_002786	ERG11	0.4987	1.004	Down

**Figure 10:**
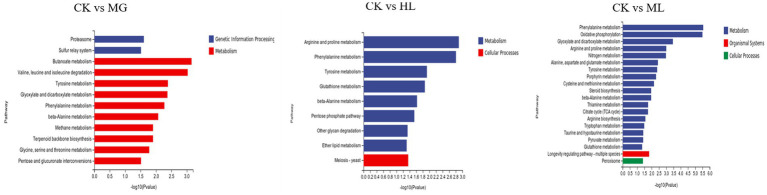
KEGG pathway enrichment analysis of differentially expressed genes in *M. furfur* treated with MG, HL, and ML.

KEGG pathway analysis showed that differentially expressed genes were enriched in pathways related to amino acid metabolism, carbohydrate metabolism, and energy metabolism, including phenylalanine metabolism, tyrosine metabolism, glycolysis, and the TCA cycle, indicating that these pathways contribute to membrane dysfunction and subsequent antifungal activity (Figure 10).

### 3.6 Effect of ML on *M. furfur* induced seborrheic dermatitis in mice

**Figure 11:**
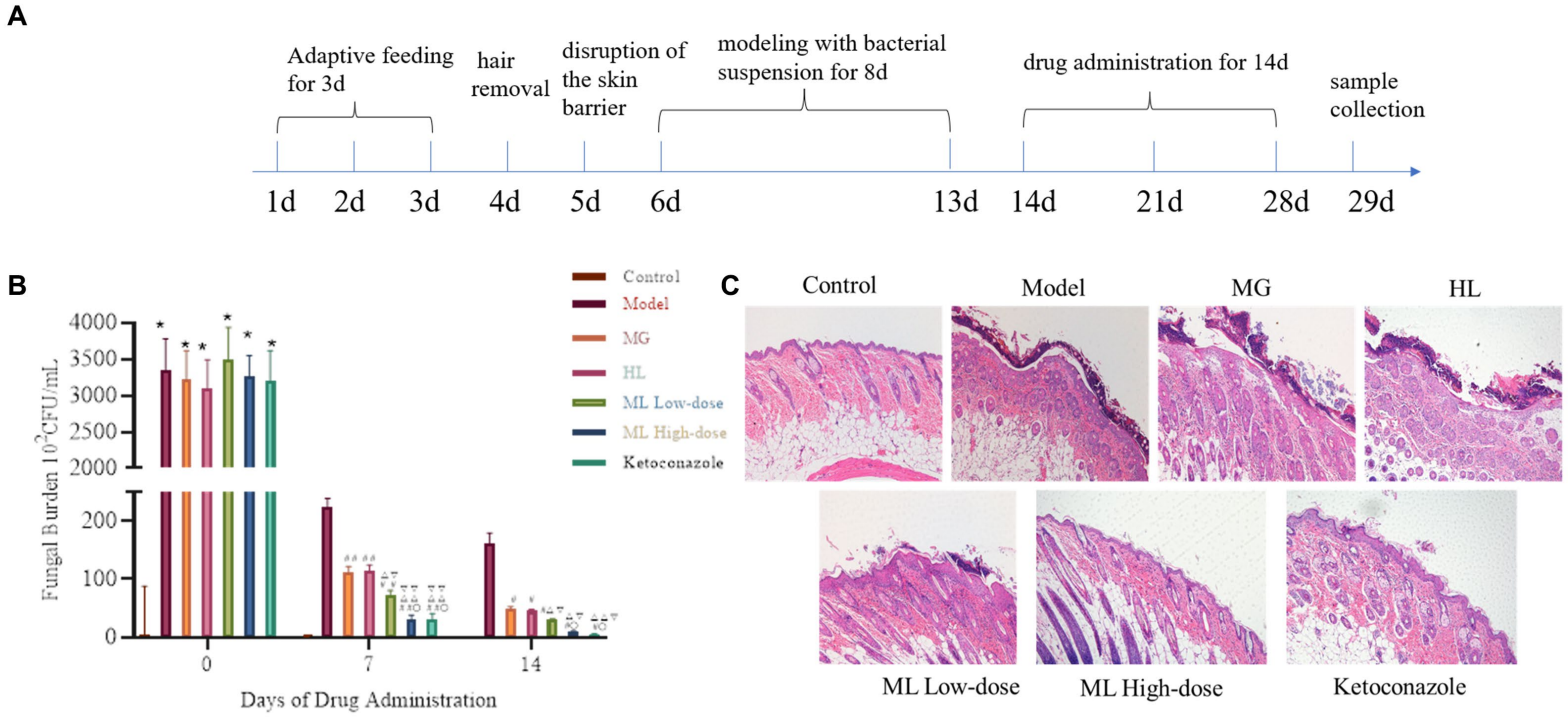
The fungal burden and the intensity of skin tissue inflammation in mice infected with *M. furfur* after the treatment of ML. **(A)** Flowchart of animal experiment; **(B)** chart of fungal burden (compared with the control, * *p* < 0.05, ** *p* < 0.01; compared with the model, ^#^*p* < 0.05, ^##^*p* < 0.01; compared with the MG, ^△^*p* < 0.05, ^△△^*p* < 0.01; compared with the HL, ^▽^*p* < 0.05, ^▽▽^*p* < 0.01; compared with the ML Lowdose, ^○^*p* < 0.05); **(C)** HE pathological sections (x10).

The seborrheic dermatitis mouse model was used to evaluate the *in vivo* synergistic antifungal efficacy of the ML. The antifungal activity of the ML was assessed through fungal colony counts and HE staining of skin lesions (Figure 11). From day 7 onwards, the fungal load on the skin of the mice treated with ML significantly decreased compared to the model group (*p* < 0.05). Additionally, the fungal load was lower in the ML groups than in the individual MG and HL groups, indicating a synergistic effect. The reduction in fungal load was dose-dependent. HE staining revealed that the model group exhibited thickened epidermis and inflammatory cell infiltration, whereas these symptoms were alleviated to varying degrees in the treatment groups. Notably, the synergistic effect of the ML observed *in vitro* was also evident *in vivo*, aligning with the reduction in fungal load and improvement in skin inflammation.

## 4 Discussion

Globally, over 140 million people suffer from *Malassezia* related skin diseases each year, significantly affecting their quality of life (Kulkarni et al., 2020). Current treatments for *Malassezia* induced skin diseases often lead to drug resistance, making the development of cost effective and efficient antifungal agents essential.

This study conducted component identification of MG and HL using HPLC, revealing their significant antifungal activity against *M. furfur* both *in vitro* and *in vivo*, with synergistic effects when used in combination, which increased with dosage. Further investigation revealed that these extracts disrupted the integrity of *M. furfur*’s cell membrane, resulting in leakage of cellular contents. Moreover, they inhibited ergosterol synthesis, which ultimately leading to cell death. The combination of these extracts showed more pronounced effects. Transcriptomic analysis indicated significant changes in genes related to the cell membrane, amino acid metabolism, carbohydrate metabolism, and energy metabolism when treated with MG and HL, either alone or in combination. Additionally, the combined use of MG and HL improved seborrheic dermatitis induced by *M. furfur*, reduced fungal load, and alleviated inflammatory conditions.

HPLC analysis, conducted by comparing with authentic standards, has confirmed the presence of specific bioactive compounds in the extracts. Specifically, MG were found to contain gallic acid and rutin, while HL contained coptisine, berberine, palmatine, and epiberberine. Our findings align with previous studies demonstrating the antibacterial properties of these compounds against various pathogens. For instance, gallic acid has been shown to inhibit *Streptococcus mutans* (Passos et al., 2021); rutin inhibits *Shigella flexneri* and *Escherichia coli* (Peng et al., 2018; Prasad et al., 2023); coptisine shows inhibitory activity against *Helicobacter pylori and Pasteurella multocida* (Tang et al., 2023; Zhang et al., 2023); berberine hydrochloride inhibits Mucor, *Candida albicans*, and *Enterococcus faecalis* (Chen et al., 2016; Aghayan et al., 2017; Huang et al., 2020); palmatine is effective against *Pseudomonas aeruginosa*, *Helicobacter pylori*, and *Staphylococcus aureus* (Aghayan et al., 2017; Long et al., 2019) and epiberberine inhibits *Helicobacter pylori* (Wu et al., 2024).

Due to the easy formation of precipitates when MG and HL are mixed, which can easily interfere with the experimental results, we chose to add a surfactant to solve this problem and enhance solubility. Since the MIC of the surfactant against *M. furfur* is greater than 250 mg/mL, we ultimately used a final concentration of 10% surfactant. The surfactant at this concentration not only effectively improved the solubility of the medication but also avoided interfering with the medication’s inhibitory activity against the fungus. According to the Clinical and Laboratory Standards Institute guidelines, the MICs of MG and HL against *M. furfur* were 12.5 mg/mL and 25 mg/mL, respectively. FICI measurements revealed a synergistic effect at concentrations of 3.125 mg/mL and 6.25 mg/mL for MG and HL, respectively, reducing the MIC by 75% when used in combination (Table 1). Additionally, time-kill curves demonstrated a dose-dependent inhibition of *M. furfur* by the ML, with combined use being more effective than individual use (Figure 2). These findings are consistent with the results of the FICI experiments. Based on the synergistic effects of the combined extracts, we further explored these extracts *in vivo* and *in vitro* antifungal mechanisms.

As a protective barrier, the cell membrane ensures the exchange of substances and energy, resisting external stress and maintaining cellular vitality and physiological responses. Maintaining membrane integrity is crucial for fungal survival. Yuan et al. (2023) found that rose essential oil inhibited cell growth by increasing the conductivity and causing leakage of proteins and nucleic acids in *Pseudomonas putida*. To elucidate the antifungal mechanism of the ML, we examined the integrity of *M. furfur*’s cell membrane after treatment. SEM and TEM observations revealed significant cell deformation, including shrinkage and wrinkling of fungal spores (Figures 3, 4). Compared to the control group, drug-treated groups showed increased protein concentration and PI-stained spores (Figure 6). These results suggest that the combination of MG and HL synergistically disrupts *M. furfur*’s cell membrane integrity, contributing to their antifungal effect.

Ergosterol, a critical component of fungal cell membranes, plays essential roles in cell physiology, determining membrane protein fluidity, permeability, and activity. Most antifungal drugs interfere with ergosterol biosynthesis or complexation, ultimately leading to cell death (Lees et al., 1995; Jordá and Puig, 2020). Zhong et al. (2022) discovered that berberine and fluconazole exhibited synergistic effects by increasing intracellular berberine concentration through ergosterol synthesis inhibition. Do et al. (2019) found that lauryl betaine affected cell membrane synthesis, particularly ergosterol, enhancing its antifungal effect. In this study, HPLC analysis demonstrated a significant reduction in ergosterol synthesis by MG and HL, with more pronounced effects when used in combination (Figure 7).

The ergosterol biosynthesis pathway is closely related to ERG genes, where ERG1 catalyzes the conversion of squalene epoxide to lanosterol, ERG7 converts lanosterol to ergosterol, and ERG11 demethylates sterols. Further steps involve ERG6 and ERG5 converting sterols to ergosterol (Liu et al., 2019). Transcriptomic analysis showed downregulation of ERG1 genes in the HL group and downregulation of ERG1, ERG5, ERG6, and ERG11 genes in the ML group (Table 3), corroborating the reduced ergosterol content observed in treated *M. furfur*. This suggests that the ML exerts its antifungal effects by downregulating multiple genes in the ergosterol synthesis pathway, disrupting cell membrane structure, and causing cellular content leakage. Interestingly, ERG6 is not involved in cholesterol synthesis, which is essential for mammalian cell membranes, indicating that targeting ERG6 could minimize host cell side effects, suggesting that the ML has the potential to become a novel, specific antifungal agent (Kodedová and Sychrová, 2015).

To further elucidate the molecular mechanisms of the ML’s antifungal activity, transcriptomic analysis revealed that MG, HL, and ML mainly inhibited *M. furfur* growth by affecting cell integrity, amino acid metabolism, carbohydrate metabolism, and energy metabolism (Figure 8). GO term enrichment showed that all three treatments impacted membrane function (Figure 9), consistent with the results of PI staining (Figure 5) and protein concentration assays (Figure 6).

Additionally, KEGG pathway enrichment analysis revealed a significant number of DEGs related to amino acid metabolism, including β-alanine, phenylalanine, tyrosine, and tryptophan metabolism (Figure 10). Amino acids serve as primary nutrients for fungi, acting as building blocks for new proteins, carbon sources, and nitrogen sources, promoting spore germination and hyphal growth, TCA metabolism, and fatty acid biosynthesis (Hildebrandt et al., 2015; Zhang et al., 2017). Some antifungal drugs interfere with amino acid metabolism and transport, inhibiting pathogen growth (Zhou et al., 2024). Besides amino acid metabolism, carbohydrate metabolism and energy metabolism, including butanoate metabolism, glycolysis, TCA cycle, and oxidative phosphorylation, were significantly affected, disrupting ATP generation and inhibiting essential life processes such as DNA replication, RNA synthesis, and protein synthesis, ultimately impairing cell membrane structure and function, inhibiting normal growth and reproduction of *M. furfur* (Pan et al., 2020).

To further investigate the *in vivo* synergistic effects of the ML against *M. furfur*, we established a seborrheic dermatitis mouse model. Compared to the model group and the individual MG and HL groups, the ML significantly reduced fungal load on the skin (*p* < 0.05), alleviating skin inflammation by reducing epidermal cell proliferation and thickness (Figure 11). These results indicate that the *in vivo* synergistic antifungal effects of the ML extract are consistent with its *in vitro* activity.

This study has established the synergistic antifungal activity of MG and HL against *M. furfur* both *in vitro* and *in vivo*, paving the way for the development of novel antifungal agents. Future work will focus on completing a comprehensive toxicity assessment of these extracts to ensure their safety and exploring their potential in various clinical applications.

## 5 Conclusion

In conclusion, the combination of MG and HL exhibits a synergistic antifungal effect against *M. furfur* in both *in vitro* and *in vivo* experiments. The synergistic effect is dose-dependent and is achieved through the inhibition of ergosterol synthesis, disruption of cell membrane integrity, and leakage of cellular contents. Transcriptomic analysis suggests that the ML affects amino acid metabolism, carbohydrate metabolism, and energy metabolism, leading to nutrient and energy deficiencies, metabolic disorders, and inhibited fungal growth and reproduction. Additionally, *in vivo* tests have demonstrated their synergistic antifungal effect by reducing fungal load, decreasing epidermal thickness, and alleviating inflammation. These findings provide a theoretical basis for understanding the antifungal mechanisms of ML and highlight its potential as a novel and specific antifungal treatment for skin diseases caused by *M. furfur*.

## Data Availability

Transcriptomic data presented in the study are deposited in the NCBI repository, accession number PRJNA1130136. The original contributions presented in the study are included in the article, further inquiries can be directed to the corresponding author.

## References

[ref1] Aghayan S. S. Mogadam H. K. Fazli M. Darban-Sarokhalil D. Khoramrooz S. S. Jabalameli F.. (2017). The effects of berberine and palmatine on efflux pumps inhibition with different gene patterns in *Pseudomonas aeruginosa* isolated from burn infections. Avicenna J. Med. Biotechnol. 9, 2–7. Available at: https://pubmed.ncbi.nlm.nih.gov/28090273, PMID: 28090273 PMC5219818

[ref2] Ayatollahi A. Firooz A. Lotfali E. Mojab F. Fattahi M. (2021). Herbal therapy for the management of seborrheic dermatitis: a narrative review. Recent Adv. Antiinfect. Drug Discov. 16, 209–226. doi: 10.2174/2772434416666211029113213 35026970

[ref3] Borda L. J. Perper M. Keri J. E. (2019). Treatment of seborrheic dermatitis: a comprehensive review. J. Dermatol. Treat. 30, 158–169. doi: 10.1080/09546634.2018.1473554, PMID: 29737895

[ref4] Boxberger M. Cenizo V. Cassir N. La Scola B. (2021). Challenges in exploring and manipulating the human skin microbiome. Microbiome 9:125. doi: 10.1186/s40168-021-01062-5, PMID: 34053468 PMC8166136

[ref5] Cendrowski A. Kraśniewska K. Przybył J. L. Zielińska A. Kalisz S. (2020). Antibacterial and antioxidant activity of extracts from rose fruits (*Rosa rugosa*). Molecules 25:1365. doi: 10.3390/molecules25061365, PMID: 32192161 PMC7144371

[ref6] Chen L. Bu Q. Xu H. Liu Y. She P. Tan R.. (2016). The effect of berberine hydrochloride on *Enterococcus faecalis* biofilm formation and dispersion in vitro. Microbiol. Res. 186-187, 44–51. doi: 10.1016/j.micres.2016.03.003, PMID: 27242142

[ref7] Chen Z. Luo T. Huang F. Yang F. Luo W. Chen G.. (2022). Kangbainian lotion ameliorates vulvovaginal candidiasis in mice by inhibiting the growth of fluconazole-resistant Candida albicans and the Dectin-1 signaling pathway activation. Front. Pharmacol. 12:816290. doi: 10.3389/fphar.2021.816290, PMID: 35140608 PMC8819624

[ref8] Chen J. Zhang J. Zhu L. Qian C. Tian H. Zhao Z.. (2022). Antibacterial activity of the essential oil from *litsea cubeba* against cutibacterium acnes and the investigations of its potential mechanism by gas chromatography-mass spectrometry metabolomics. Front. Microbiol. 13:823845. doi: 10.3389/fmicb.2022.823845, PMID: 35308342 PMC8924494

[ref9] Do E. Lee H. G. Park M. Cho Y.-J. Kim D. H. Park S.-H.. (2019). Antifungal mechanism of action of lauryl betaine against skin-associated fungus *Malassezia restricta*. Mycobiology 47, 242–249. doi: 10.1080/12298093.2019.1625175, PMID: 31448144 PMC6691833

[ref10] Grice E. A. Segre J. A. (2011). The skin microbiome. Nat. Rev. Microbiol. 9:244253, 244–253. doi: 10.1038/nrmicro2537 PMC3535073 21407241

[ref11] Hamdino M. Saudy A. A. El-Shahed L. H. Taha M. (2022). Identification of Malassezia species isolated from some Malassezia associated skin diseases. J. Med. Mycol. 32:101301. doi: 10.1016/j.mycmed.2022.101301, PMID: 35700659

[ref12] Hildebrandt T. M. Nesi A. N. Araújo W. L. Braun H.-P. (2015). Amino acid catabolism in plants. Mol. Plant 8, 1563–1579. doi: 10.1016/j.molp.2015.09.005, PMID: 26384576

[ref13] Huang X. Lu J. Xing S. Sun L. (2021). Research progress on toxicology and risk assessment of zinc pyrithione as cosmetics ingredient. China Surfactant Deterg. Cosmet. 51, 1235–1241.

[ref14] Huang X. Zheng M. Yi Y. Patel A. Song Z. Li Y. (2020). Inhibition of berberine hydrochloride on *Candida albicans* biofilm formation. Biotechnol. Lett. 42, 2263–2269. doi: 10.1007/s10529-020-02938-6, PMID: 32557120

[ref15] Ianiri G. LeibundGut-Landmann S. Dawson T. L. Jr. (2022). Malassezia: a commensal, pathogen, and mutualist of human and animal skin. Ann. Rev. Microbiol. 76, 757–782. doi: 10.1146/annurev-micro-040820-010114, PMID: 36075093

[ref16] Jordá T. Puig S. (2020). Regulation of ergosterol biosynthesis in *Saccharomyces cerevisiae*. Genes 11:795. doi: 10.3390/genes11070795, PMID: 32679672 PMC7397035

[ref17] Kodedová M. Sychrová H. (2015). Changes in the sterol composition of the plasma membrane affect membrane potential, salt tolerance and the activity of multidrug resistance pumps in *Saccharomyces cerevisiae*. PLoS One 10:e0139306. doi: 10.1371/journal.pone.0139306, PMID: 26418026 PMC4587746

[ref18] Kong W. J. Zhao Y. L. Xiao X. H. Li Z. L. Jin C. Li H. B. (2009). Investigation of the anti-fungal activity of coptisine on *Candida albicans* growth by microcalorimetry combined with principal component analysis. J. Appl. Microbiol. 107, 1072–1080. doi: 10.1111/j.1365-2672.2009.04292.x, PMID: 19426275

[ref19] Kulkarni M. Hastak V. Jadhav V. Date A. A. (2020). Fenugreek leaf extract and its gel formulation show activity against Malassezia furfur. Assay Drug Dev. Technol. 18, 45–55. doi: 10.1089/adt.2019.918, PMID: 31524496 PMC6998042

[ref20] Lees N. Skaggs B. Kirsch D. Bard M. (1995). Cloning of the late genes in the ergosterol biosynthetic pathway of *Saccharomyces cerevisiae*—a review. Lipids 30, 221–226. doi: 10.1007/BF02537824, PMID: 7791529

[ref21] Liu J.-F. Xia J.-J. Nie K.-L. Wang F. Deng L. (2019). Outline of the biosynthesis and regulation of ergosterol in yeast. World J. Microbiol. Biotechnol. 35, 1–8. doi: 10.1007/s11274-019-2673-2 31222401

[ref22] Liu B.-G. Xie M. Dong Y. Wu H. He D.-D. Hu G.-Z.. (2022). Antimicrobial mechanisms of traditional Chinese medicine and reversal of drug resistance: a narrative review. Eur. Rev. Med. Pharmacol. Sci. 26, 5553–5561. doi: 10.26355/eurrev_202208_29426 35993652

[ref23] Long J. Song J. Zhong L. Liao Y. Liu L. Li X. (2019). Palmatine: a review of its pharmacology, toxicity and pharmacokinetics. Biochimie 162, 176–184. doi: 10.1016/j.biochi.2019.04.008, PMID: 31051209

[ref24] Nowak R. Olech M. Pecio Ł. Oleszek W. Los R. Malm A.. (2014). Cytotoxic, antioxidant, antimicrobial properties and chemical composition of rose petals. J. Sci. Food Agric. 94, 560–567. doi: 10.1002/jsfa.6294, PMID: 23818393

[ref25] OuYang Q. Liu Y. Oketch O. R. Zhang M. Shao X. Tao N. (2021). Citronellal exerts its antifungal activity by targeting ergosterol biosynthesis in *Penicillium digitatum*. J. Fungi 7:432. doi: 10.3390/jof7060432, PMID: 34072578 PMC8229684

[ref26] Pan C. Li Y.-X. Yang K. Famous E. Ma Y. He X.. (2020). The molecular mechanism of perillaldehyde inducing cell death in aspergillus flavus by inhibiting energy metabolism revealed by transcriptome sequencing. Int. J. Mol. Sci. 21:1518. doi: 10.3390/ijms21041518, PMID: 32102190 PMC7073185

[ref27] Passos M. R. Almeida R. S. Lima B. O. Rodrigues J. Z. S. Macedo Neres N. S. Pita L. S.. (2021). Anticariogenic activities of Libidibia ferrea, gallic acid and ethyl gallate against *Streptococcus mutans* in biofilm model. J. Ethnopharmacol. 274:114059. doi: 10.1016/j.jep.2021.114059, PMID: 33794333

[ref28] Peng L. Y. Yuan M. Cui Z. Q. Wu Z. M. Yu Z. J. Song K.. (2018). Rutin inhibits quorum sensing, biofilm formation and virulence genes in avian pathogenic *Escherichia coli*. Microb. Pathog. 119, 54–59. doi: 10.1016/j.micpath.2018.04.007, PMID: 29627449

[ref29] Prasad R. S. Chikhale R. V. Rai N. Akojwar N. S. Purohit R. A. Sharma P.. (2023). Rutin from Begonia roxburghii modulates iNOS and Sep a activity in treatment of *Shigella flexneri* induced diarrhoea in rats: An in vitro, in vivo and computational analysis. Microb. Pathog. 184:106380. doi: 10.1016/j.micpath.2023.106380, PMID: 37821049

[ref30] Schwartz J. R. (2016). Zinc Pyrithione: a topical antimicrobial with complex pharmaceutics. J. Drugs Dermatol. 15, 140–144. 26885780

[ref31] Tang Q. Ma Z. Tang X. Liu Y. Wu H. Peng Y.. (2023). Coptisine inhibits helicobacter pylori and reduces the expression of CagA to alleviate host inflammation in vitro and in vivo. J. Ethnopharmacol. 314:116618. doi: 10.1016/j.jep.2023.116618 37164257

[ref32] Vest B. E. Krauland K. (2023). "Malassezia furfur," in StatPearls. St. Petersburg, FL: StatPearls Publishing. 31971731

[ref33] Wang Y. An H. Guo Y.-N. Wang Q. Shang Y.-Y. Chen M.-K.. (2023). Anthocyanins from Malus spp. inhibit the activity of Gymnosporangium yamadae by downregulating the expression of WSC, RLM1, and PMA1. Front. Microbiol. 14:1152050. doi: 10.3389/fmicb.2023.1152050, PMID: 37206329 PMC10191115

[ref34] Wang L. Li H. Chen J. Wang Y. Gu Y. Jiu M. (2024). Antibacterial mechanisms and Antivirulence activities of Oridonin against pathogenic *Aeromonas hydrophila* AS 1.1801. Microorganisms 12:415. doi: 10.3390/microorganisms12020415, PMID: 38399819 PMC10891661

[ref35] Wang J. Wang L. Lou G.-H. Zeng H.-R. Hu J. Huang Q.-W.. (2019). Coptidis Rhizoma: a comprehensive review of its traditional uses, botany, phytochemistry, pharmacology and toxicology. Pharm. Biol. 57, 193–225. doi: 10.1080/13880209.2019.1577466, PMID: 30963783 PMC6461078

[ref36] Wang S. Xu M. Han Y. Zhou Z. (2024). Exploring mechanisms of antifungal Lipopeptide Iturin a from Bacillus against aspergillus Niger. J. Fungi 10:172. doi: 10.3390/jof10030172, PMID: 38535181 PMC10970988

[ref37] White R. L. Burgess D. S. Manduru M. Bosso J. A. (1996). Comparison of three different in vitro methods of detecting synergy: time-kill, checkerboard, and E test. Antimicrob. Agents Chemother. 40, 1914–1918. doi: 10.1128/AAC.40.8.1914, PMID: 8843303 PMC163439

[ref38] Wu H. Xie X. Tang Q. Huang T. Tang X. Jiao B.. (2024). Epiberberine inhibits helicobacter pylori and reduces host apoptosis and inflammatory damage by down-regulating urease expression. J. Ethnopharmacol. 318:117046. doi: 10.1016/j.jep.2023.117046, PMID: 37586440

[ref39] Yang X. Xiong B. Yuan Z. Liao H. Liu X. Wu Y.. (2022). Polygalaxanthone III, an active ingredient in *Polygala japonica Houtt.*, repaired *Malassezia*-stimulated skin injury via STAT3 phosphorylated activation. Molecules 27:7520. doi: 10.3390/molecules27217520 36364345 PMC9655589

[ref40] Yuan Y. Liu L. Guo L. Wang L. Liu Y. (2023). Antibacterial mechanism of rose essential oil against *Pseudomonas putida* isolated from white Hypsizygus marmoreus at cellular and metabolic levels. Ind. Crop. Prod. 196:116523. doi: 10.1016/j.indcrop.2023.116523

[ref41] Zhang R. Tian S. Zhang T. Zhang W. Lu Q. Hu Q.. (2023). Antibacterial activity mechanism of coptisine against *Pasteurella multocida*. Front. Cell. Infect. Microbiol. 13:1207855. doi: 10.3389/fcimb.2023.1207855, PMID: 37502603 PMC10369072

[ref42] Zhang S. Zeng X. Ren M. Mao X. Qiao S. (2017). Novel metabolic and physiological functions of branched chain amino acids: a review. J. Anim. Sci. Biotechnol. 8, 1–12. doi: 10.1186/s40104-016-0139-z 28127425 PMC5260006

[ref43] Zhong H. Han L. Lu R.-Y. Wang Y. (2022). Antifungal and immunomodulatory ingredients from traditional Chinese medicine. Antibiotics 12:48. doi: 10.3390/antibiotics12010048, PMID: 36671249 PMC9855100

[ref44] Zhou T. Pan J. Wang J. Yu Q. Zhang P. Lai T. (2024). Inhibitory properties of cinnamon bark oil against postharvest pathogen Penicillium digitatum in vitro. J. Fungi 10:249. doi: 10.3390/jof10040249, PMID: 38667920 PMC11051492

